# Proton spectroscopic imaging of brain metabolites in basal ganglia of healthy older adults

**DOI:** 10.1007/s10334-014-0465-0

**Published:** 2014-10-14

**Authors:** Jehill Parikh, Michael J. Thrippleton, Catherine Murray, Paul A. Armitage, Bridget A. Harris, Peter J. D. Andrews, Joanna M. Wardlaw, John M. Starr, Ian J. Deary, Ian Marshall

**Affiliations:** 1 Centre for Clinical Brain Sciences, University of Edinburgh, Chancellor’s Building, Edinburgh, EH16 4SB UK; 2 Department of Psychology, University of Edinburgh, Edinburgh, UK; 3 Academic Unit of Radiology, Department of Cardiovascular Science, University of Sheffield, Sheffield, UK; 4 Critical Care Medicine, NHS Lothian and University of Edinburgh, Edinburgh, UK; 5 Centre for Cognitive Ageing and Cognitive Epidemiology, University of Edinburgh, Edinburgh, UK

**Keywords:** MR spectroscopy, Brain metabolites, Basal ganglia, Ageing, Older adults, Healthy volunteers

## Abstract

**Object:**

We sought to measure brain metabolite levels in healthy older people.

**Materials and methods:**

Spectroscopic imaging at the level of the basal ganglia was applied in 40 participants aged 73–74 years. Levels of the metabolites *N*-acetyl aspartate (NAA), choline, and creatine were determined in "institutional units" (IU) corrected for T1 and T2 relaxation effects. Structural imaging enabled determination of grey matter (GM), white matter (WM), and cerebrospinal fluid content. ANOVA analysis was carried out for voxels satisfying quality criteria.

**Results:**

Creatine levels were greater in GM than WM (57 vs. 44 IU, *p* < 0.001), whereas choline and NAA levels were greater in WM than GM [13 vs. 10 IU (*p* < 0.001) and 76 versus 70 IU (*p* = 0.03), respectively]. The ratio of NAA/cre was greater in WM than GM (2.1 vs. 1.4, *p* = 0.001) as was that of cho/cre (0.32 vs. 0.16, *p* < 0.001). A low voxel yield was due to brain atrophy and the difficulties of shimming over an extended region of brain.

**Conclusion:**

This study addresses the current lack of information on brain metabolite levels in older adults. The normal features of ageing result in a substantial loss of reliable voxels and should be taken into account when planning studies. Improvements in shimming are also required before the methods can be applied more widely.

## Introduction

The study of normal brain ageing, neurodegenerative conditions, and age-related cognitive decline is of increasing interest as populations age. Magnetic resonance imaging is proving to be a powerful tool for the evaluation of structural and functional changes [1, 2]. Additionally, magnetic resonance spectroscopy (MRS) can provide information on brain metabolites such as N-acetyl aspartate (NAA), choline, creatine, and glutamate. Since metabolites are related to cellular function, it is understandable that brain metabolites have been studied for their associations with cognitive function.

Although brain metabolite levels have been measured in modest groups of young healthy subjects, there is relatively little literature concerning older adults. As early as 1993, Christiansen et al. [3] measured metabolite levels and relaxation times in healthy young adults (*n* = 8; age 20–30) compared with healthy elderly adults (*n* = 8; age 60–80). They found a 15 % decline in occipital NAA in the older group compared with the younger group. In a recent meta-analysis of the available literature, Haga et al. [4] reported reduced NAA and raised choline and creatine in healthy older subjects (>60 years) relative to healthy younger subjects (<60 years). Previously, we used a single-voxel MRS technique and found an association between raised creatine and a decline in cognitive ability [5]. Kantarci et al. [6] reported a decreased NAA/creatine ratio in people with mild cognitive impairment and Alzheimer's disease relative to cognitively normal, age-matched controls.

Magnetic resonance spectroscopic imaging (MRSI) enables measurement of multiple voxels in a slice across the brain and thereby opens up the possibility of producing maps of metabolite distribution.

The objective of the current study was to establish brain metabolite levels in healthy older adults within a narrow age range. We employed strict criteria to discard spectroscopic voxels that failed various quality tests. We looked for associations between metabolite levels, brain atrophy, and cognitive scores.

## Materials and methods

### Participants

Forty participants (25 female) were recruited from healthy, community-living people comprising the Lothian Birth Cohort 1936 (LBC 1936 [7, 8]). All participants were aged 73 or 74 at the time of MRI scanning and formed a sub group of approximately 650 volunteers who were part of a longitudinal study on cognitive ageing [7] and for whom recent detailed structural imaging [9] and cognitive scores were available. The latter included general fluid intelligence [10] and general memory [11]. An experienced observer determined brain atrophy, iron, and white matter (WM) lesion scores [12] from the structural images. Participants with the lowest ratings were then selected for the present spectroscopic study . The study was approved by the local research ethics committee and all participants gave informed written consent.

In order to avoid any possible confounding effects from diurnal variation, all scans were performed at the same time of day (between 11 a.m. and 1 p.m.).

### Imaging

All imaging was performed on a 1.5T clinical research scanner (HDxt, GE Healthcare, UK). The imaging protocol for this spectroscopy study used a standard quadrature head coil and included localiser, coronal T1-weighted (T1w: 3D-IR-PREP-FAST-GRE), and axial T2-weighted (T2w: FSE) scans in addition to an MRSI scan for brain metabolite measurements. Key acquisition parameters for the T1w scans were 3D inversion recovery-prepared, TI/TR/TE = 500/10/4 ms, flip angle 8°, matrix 192 × 192, FOV 256 mm slices of 1.3 mm thickness (156 slices total). Parameters for the T2w scans were TR/TE = 4,000/102 ms, matrix 256 × 256, FOV 240 mm, slices of 5 mm thickness (28 slices total), and 2 signal averages.

**Fig. 1:**
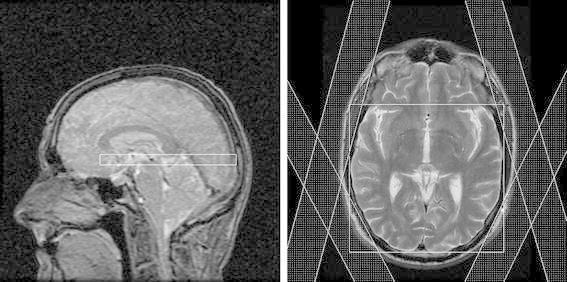
Sagittal and axial localiser scan showing placement of PRESS–MRSI slab and additional saturation bands (hatched) to minimise lipid contamination

The T2w images were used to plan a 10 mm thick axial MRSI slice at a level that included the basal ganglia. PRESS excitation was used with a volume of interest (VOI) selected to cover the brain laterally and extend forward from the posterior edge of the brain, avoiding the frontal sinuses and orbits (Fig. 1). 24 × 24 phase-encoding steps were applied over a field of view of 240 mm. Repetition and echo times were 1,000 and 145 ms, respectively. For each phase encode, 512 complex data points were acquired with a sampling interval of 1 ms. Manufacturer-provided (first order) automatic shimming was employed to reduce the B0 magnetic field inhomogeneity prior to the MRSI acquisitions. Water suppression used the default CHESS technique with three selective RF pulses. Outer volume suppression employed pre-saturation slabs parallel to the faces of the VOI and an additional four slabs positioned around the edges of the brain to suppress lipid artifacts from the scalp (Fig. 1). The total examination time was approximately 30 min.

### Image analysis

MRSI data were processed offline as described previously [13]. Briefly, a two-dimensional spatial Fourier Transform with cosine-weighted apodisation was applied to the raw spectroscopic data, followed by zero-order phase and eddy-current correction on a voxel-by-voxel basis using the residual water signals, bringing water to a fixed nominal chemical shift of 4.70 ppm [14]. The residual water component was removed from each signal using the Hankel Lanczos singular value decomposition technique and the resulting signals quantified using the AMARES algorithm within the MRUI package (www.mrui.uab.es). A model consisting of three in-phase Gaussian components (corresponding to the major metabolite peaks of choline, creatine, and NAA) and two inverted components (corresponding to lactate) was used. Spectra were automatically discarded if fitted line widths were <1 Hz or >10 Hz; if the metabolite peaks were more than 0.1 ppm offset from their nominal values; or if the voxels lay on the edges of the PRESS excitation region. All spectra were also inspected visually and discarded if judged to be of poor quality, for example having a badly elevated baseline or containing spurious peaks. Spectral peak areas were corrected for relaxation effects using literature values for T1 and T2 in basal ganglia in elderly subjects [3] and normalised according to the number of contributing protons. The resulting institutional units are directly related to molecular concentrations and enable comparison between studies.

Tissue segmentation maps for grey matter (GM), WM, cerebrospinal fluid (CSF), and “background” (BGND) were computed from the T1-weighted images using the FSL-FAST algorithm (http://www.fmrib.ox.ac.uk). Previously available T2* (gradient-echo) images [9] were first registered with the T1w and T2w images and used to create brain masks. Air and non-brain tissue outside the mask was classed as BGND. Tissue segmentation maps were then generated and overlaid on the MRSI grid. For each spectroscopic voxel, the percentage of GM, WM, CSF, and BGND content was calculated based on three contiguous slices to match the extent of the MRSI acquisition. Spectroscopy voxels containing more than 20 % CSF or 5 % BGND were rejected.

ANOVA (Matlab) and linear mixed effects (PASW Statistics) analyses were carried out on metabolite levels, with (%WM–%GM) as a variable and "subject" as a random factor. Metabolite ratios were also analysed in the same way. Subject-by-subject associations between metabolite levels and structural and cognitive scores were explored.

## Results

Imaging and automatic brain segmentation succeeded in all 40 participants. Three participants who had fewer than five spectroscopic voxels valid for NAA, choline, or creatine were excluded from the subject-by-subject analysis.

### Metabolite levels

**Fig. 2:**
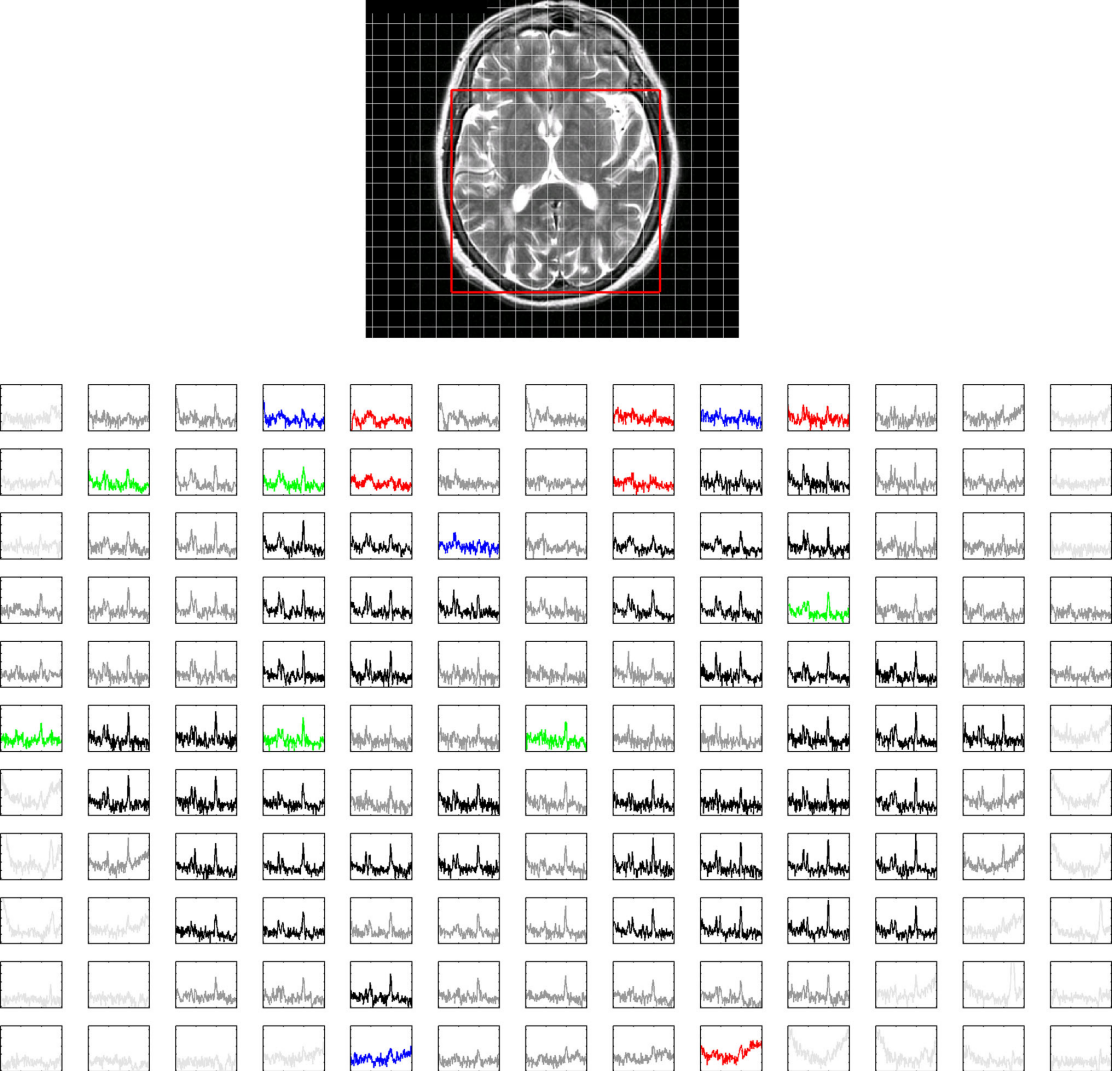
MRSI grid superimposed on corresponding T2-weighted image. The PRESS *excitation box* is outlined in *red*. Below is shown the grid of spectra contained completely within the *PRESS box*, colour coded according to: *pale grey*, ‘background’ (BGND), where brain comprises <95 % of the voxel volume; *grey*, CSF more than 20 %; *red*, fitting of NAA peak failed; *blue*, failed visual quality test; *green*, failed choline and/or creatine fitting but valid for NAA; *black*, passed all tests. Tests were applied in the order described. For further details, refer to text. The spectral range displayed is from 4 to 1 ppm

**Fig. 3:**
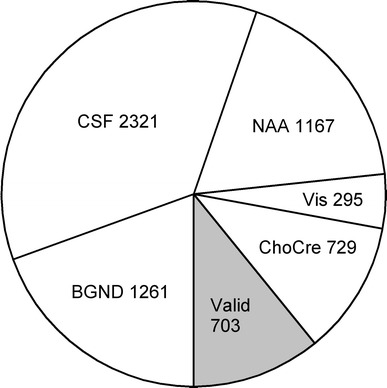
Exclusion of spectroscopic voxels for the cohort. 40 subjects had a potential 6,476 voxels, of which 1,432 were valid for NAA after spectral quality tests had been applied. Of these, 703 voxels were also valid for choline and creatine measurement. Reasons for exclusion were: background more than 5 % of voxel volume (BGND); CSF more than 20 % (CSF); fitting of NAA peak failed (NAA); failed visual quality test (Vis); failed choline and/or creatine fitting (ChoCre), but valid for NAA. Tests were applied in the order described, and the number of additional voxels failing each test is given in the figure

Of the combined total of 6,476 voxels contained within the MRSI PRESS excitation regions (Fig. 1), 1,432 survived the spectral tests for NAA, and only 703 survived when including choline and creatine. These 703 voxels were included in the full ANOVA analyses reported in Table 1. Figures 2 and 3 display the reasons for this attrition. There was a significant negative correlation between the percentage of valid voxels and the mean NAA line width (of retained voxels) for each subject (*r* = −0.44, *p* = 0.005). The main metabolite findings are that creatine levels were significantly higher in GM than WM, whereas choline and NAA levels were significantly higher in WM than GM. Choline and creatine levels were highly correlated (*r* = 0.55, *p* < 0.001), whilst creatine and NAA showed a weak negative correlation (*r* = −0.32, *p* = 0.05). NAA/cre and cho/cre metabolite ratios were significantly greater in WM than GM. The NAA/cho ratio was greater in GM than WM, although this difference did not quite reach significance (*p* = 0.06).

**Table 1:** Metabolite levels [in institutional units (IU): see text] and metabolite ratios from ANOVA analysis of spectroscopic voxels in 40 subjects

	WM	GM	*p* (GM vs WM)
Metabolites (IU)			
NAA	76	70	0.03
Choline	13	10	<0.001
Creatine	45	57	<0.001
Metabolite ratios			
NAA/Cho	8.2	9.8	0.06
NAA/Cre	2.1	1.4	<0.001
Cho/Cre	0.32	0.16	<0.001
Spectral peak area ratios			
NAA/Cho	2.4	2.8	0.06
NAA/Cre	2.4	1.6	<0.001
Cho/Cre	1.3	0.7	<0.001

Ratios of spectral peak areas are also given for comparison with other studies. Only voxels passing all spectral quality tests (see Figs. 2, 3 and text) are included

*WM* white matter, *GM* grey matter

### Relation to structural and cognitive scores

There was a trend for choline levels to be associated with WM lesion scores (*r* = 0.24, *p* = 0.1). There were no associations between metabolites and either atrophy or iron deposition scores. Creatine levels were correlated with fluid intelligence (*r* = 0.33, *p* = 0.05), and NAA showed a trend towards association with memory scores (*r* = 0.27, *p* = 0.1).

## Discussion

In this study we used MRSI to measure brain metabolite levels in healthy elderly subjects. We looked for evidence of tissue type differences and for associations with structural and cognitive scores. The narrow age range of the Lothian Birth Cohort 1936 removes age as a potential confounding factor, although it does preclude looking for age-related effects in a single study such as this.

Our finding of higher creatine levels in GM than WM is in agreement with previous literature reports, although the magnitude of the difference varies greatly, being 27 % in the present study, 11 % in Schuff et al. [15], 30 % in Hetherington et al. [16], 40 % in Tal et al. [17], and as much as 70 % in McLean et al. [18]. Our finding of lower NAA levels in GM than in WM (70 vs. 76 IU) is consistent with some previous reports [15, 16] but different from others. For example, Tal et al. [17] averaged metabolite values over a large centrally placed volume of brain and found that NAA was 11 % higher in GM than WM. We found choline levels lower in GM than WM, consistent with previous reports [16, 18], although others have reported no significant difference [15, 17]. Christiansen et al. [3] found no difference in metabolite levels between basal ganglia and the occipital, temporal, and frontal regions in healthy elderly subjects. However, they used large (8 mL) volumes of interest containing unspecified proportions of GM and WM. Our results for metabolite ratios are consistent with our findings for individual metabolite levels. The reasons for the discrepancies in metabolite findings between various studies are unclear but will include exact details of the positioning, acquisition method and quantification, and possible (unknown) differences in metabolite T1 and T2 values between GM and WM.

We used literature values for T1 and T2 correction and also normalised the results by the number of protons contributing to the spectral peaks (3 for NAA and creatine, 9 for choline). The final results in institutional units are therefore closely related to molecular concentrations. Other researchers, including [3] for example, have reported ratios of spectral peak areas under experimental conditions, and we have included these ratios in Table 1 to enable comparisons with other studies. We did not explore regional variations because of the relatively low number of valid voxels in particular regions, especially the frontal and cortical regions. The chemical shift displacement between choline and NAA amounted to approximately 0.5 mm through-plane (i.e. 5 % of the slab thickness), 4 mm anterior-posterior, and 6 mm left–right. Since we excluded voxels around the edge of the excitation volume, these displacements will have had minimal effect.

In this study we found a positive correlation between creatine and fluid intelligence score, whereas previously [5] we found a negative correlation between parietal cortex creatine and memory scores but no association with other cognitive measures. However, the two studies investigated different brain regions.

As in recent work by Bainbridge et al. [19], we employed strict criteria to discard spectroscopic voxels that failed various quality tests. Although rather conservative, such criteria can be applied with minimal human intervention, will become important for future clinical studies, and will be essential when large data sets from whole-brain spectroscopy [20] become routinely available. The large loss of voxels (Fig. 3) was caused by a high proportion of CSF and failures of spectral quantification. In older people with brain atrophy, it is inevitable that voxels will be lost due to CSF and enlarged ventricles. Failures of quantification were due in part to the problems of shimming over an extended region of the brain, which is especially difficult at the level of the basal ganglia. In some subjects, an unavoidable tilt of the head relative to the scanner led to portions of the frontal sinuses, ethmoid sinus, or eyes being included in the MRSI plane. This is a particular problem in older people, as they are less able to tilt their heads into the optimum posture. Only first-order (linear) shimming was available on the scanner used. In other studies (data not shown) we have found greater success rates with MRSI slices at the level of the corpus callosum. The use of modern scanners with higher-order shims is likely to help, as are higher field strengths and more sensitive multi-channel coils enabling higher SNR and hence smaller voxels with improved line widths. However, some brain areas, for example the frontal and temporal regions, will remain challenging in this regard.

A limitation of this study is that time did not permit the collection of non-suppressed spectroscopic data for water referencing purposes. Instead we used the residual water signal for "self-referencing" [14]. Ideally, a rapid interleaved (suppressed/reference) technique would be used, as demonstrated by the echo-planar acquisition scheme of Ebel and Maudsley [20].

## Conclusions

The measurement of brain metabolites is potentially useful in studies of healthy ageing and acute and neurodegenerative diseases. There is currently a lack of information on metabolite levels in older adults. The current study of a moderately sized cohort with a tight age range adds to the available literature. However, technical challenges such as shimming across the whole brain and water referencing need to be resolved before the technique can be more widely applied in large trials. Future directions include rapid, three-dimensional, whole-brain coverage and longitudinal studies.

## Abbreviations

BGND: Background (non-brain tissue and air)
CHESS: Chemical shift selective
CSF: Cerebrospinal fluid
GM: Grey matter
LBC: Lothian Birth Cohort
NAA: *N*-acetyl aspartate
PRESS: Point-resolved spectroscopy
WM: White matter
